# Novel Janus gamma-Pb$_2$XY monolayers with high thermoelectric performance X=S, Se and Y=Se, Te X$\ne$Y

**DOI:** 10.1038/s41598-024-67039-0

**Published:** 2024-07-19

**Authors:** Efracio Mamani Flores, Victor José Ramirez Rivera, Fredy Mamani Gonzalo, Jose Ordonez-Miranda, Julio R. Sambrano, Mario Lucio Moreira, Maurício Jeomar Piotrowski

**Affiliations:** 1 Department of Physics, Jorge Basadre Grohmann National University, Tacna, Perú; 2 Department of Physics, Federal University of Pelotas, Pelotas, Rio Grande do Sul Brazil; 3 LIMMS, CNRS-IIS IRL 2820, The University of Tokyo, Tokyo, 153-8505 Japan; 4 Modeling and Molecular Simulation Group, São Paulo State University, Bauru, São Paulo, 17033-360 Brazil

**Keywords:** Condensed-matter physics, Materials for energy and catalysis, Theory and computation

## Abstract

The quest for efficient thermoelectric materials has intensified with the advent of novel Janus monolayers exhibiting exceptional thermoelectric parameters. In this work, we comprehensively investigate the structural, electronic, transport, phonon, and thermoelectric properties of novel Janus $\gamma$-Pb$_2$XY (X=S, Se; Y=Se, Te; X$\ne$Y) monolayers using density functional theory combined with the Boltzmann transport equation. Our findings unveil the energetic, dynamic, thermal, and mechanical stability of these monolayers, along with their remarkable thermoelectric performance. Remarkably, the *p*-type $\gamma$-Pb$_2$SeTe monolayer exhibits an outstanding figure of merit (*ZT*) of 6.88 at 800 K, attributed to its intrinsically low lattice thermal conductivity of 0.162 Wm$^{-1}$K$^{-1}$ arising from strong phonon scattering, low group velocity, low phonon relaxation time, and a high Grüneisen parameter. Furthermore, these monolayers demonstrate high Seebeck coefficients and electrical conductivities, making them promising for efficient charge transport and thermoelectric energy conversion. Our results highlight the immense potential of Janus $\gamma$-Pb$_2$XY monolayers as promising candidates for high-temperature thermoelectric applications and open up exciting avenues for further exploration of these novel two-dimensional materials in energy-related technologies.

## Introduction

In recent years, two-dimensional (2D) materials have attracted significant attention due to their enhanced physical properties compared to the corresponding bulk counterparts^1,2^. These promising materials have a wide range of applications, including electronics^3^, optoelectronics^4^, photovoltaics^5^, gas detection^6^, and thermoelectricity^7^.

For instance, the recent synthesis of bulk $\gamma$-GeSe via chemical vapor deposition (CVD)^8^ has prompted further investigation exploring its thermoelectric properties under monolayers form^9^. The $\gamma$ phase of group IV monochalcogenide is characterized by a buckled honeycomb lattice with a four-atomic-thick layer, presenting intriguing possibilities for its layered structure and corresponding Van der Waals (vdW) interlayer interactions involved. Notably, Jia et al.^10^ reported that $\gamma$-PbX monolayers exhibit a low lattice thermal conductivity and a high thermoelectric figure of merit *ZT* ranging from 1.97 to 2.45, under a $4\%$ of strain. Further, the recent synthesis of Janus monolayers by sulfurization^11^ and remote hydrogen plasma^12^, through the replacement of a top layer with another chemical compound, typically from the same family, enables to break the symmetry and tailor the properties of non-Janus base monolayers. In particular, this symmetry break via Janus monolayers has the potential to enhance the thermoelectric response of materials, however it is not explored yet.

The thermoelectric performance of materials is determined by the following well-known figure of merit

1 $$\begin{aligned} ZT = \frac{\sigma S^2}{\kappa _e+\kappa _l}T= \frac{PF}{\kappa _e+\kappa _l}T, \end{aligned}$$

where $\sigma$ is the electrical conductivity, *S* is the Seebeck coefficient, $PF =\sigma S^2$ is the power factor, *T* is the temperature, and $\kappa _e$ and $\kappa _l$ are the electron and phonon thermal conductivities, respectively. A thermoelectric material with high rate of heat-to-electricity conversion therefore should exhibit high Seebeck coefficient, high electrical conductivity, and low thermal conductivity. As there is a strong correlation among these three physical properties, the maximization of *ZT* is challenging. For instance, materials with a wide bandgap tend to have a high Seebeck coefficient, but a low electrical conductivity, which reduces *PF* and hence *ZT*. Another approach to maximize *ZT* involves searching for materials with low lattice thermal conductivity, which can be achieved through strain engineering^13–15^, phonon manipulation^16–18^, including four-phonon processes^19^ or doping^20,21^. Considering that $\kappa _e$ is proportional to $\sigma$, the reduction of $\kappa _l$ represents a key strategy to enhance *ZT*^22^.

In this study, we systematically investigate the thermoelectric properties of Janus $\gamma$-Pb$_2$XY (X$=$S, Se; Y$=$Se, Te; X$\ne$Y) monolayers using density functional theory (DFT) combined with the Boltzmann transport equation (BTE). We find a notable reduction in the cross-plane lattice thermal conductivity of all Janus monolayers and explain the physical mechanisms driving its values. A maximum figure of merit $ZT=6.88$ was obtained for *p*-type carriers at 800 K for Janus $\gamma$-Pb$_2$SeTe monolayer. These findings highlight the exceptional thermoelectric response of Janus $\gamma$-Pb$_2$XY monolayers and lay the foundation for their applications in thermoelectricity.

## Computational details

Theoretical calculations were conducted using the open-access software Quantum Espresso^23^ employing the projector-augmented wave (PAW) method^24^ with the Perdew–Burke–Ernzerhof (PBE) formulation^25^ for the structural, mechanical, electronic and thermoelectric properties of this research. To mitigate overestimations of electronic properties, we employed the Heyd–Scuseria–Ernzerhof (HSE06) hybrid functional^26^. van der Waals interactions within each Janus monolayer were accounted for using the DFT-D3 approach by Grimme^27^. Brillouin zone integration was performed using an 18x18x1 **k**-point grid, with a cut-off energy of 800 eV for the expansion of the plane wave basis. Convergence criteria for structural optimization were set to a force threshold of $1 \times 10^{-3}$ Ry/Bohr, with an energy convergence criterion of $10^{-12}$ Ry for self-consistent electronic iterations. Convergence tests, including those for the energy cutoff and **k**-mesh, are detailed in the Supplementary Material (SM) (Fig. S1).

**Figure 1:**
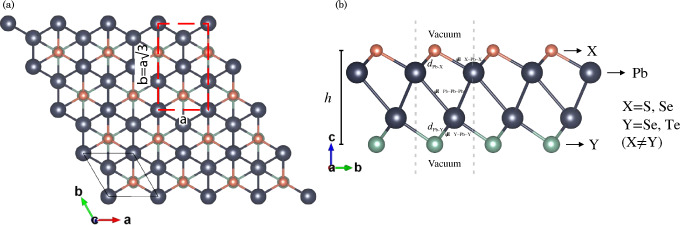
(**a**) Top and (**b**) side views of Janus $\gamma$-Pb$_2$XY (X$=$S, Se; Y$=$Se, Te; X$\ne$Y) monolayers. In (**a**), the solid black line represents the unit cell, while the red dashed line denotes the orthogonal cell used for calculating the elastic constants. Figure (**b**) presents a schematic diagram illustrating the distances, angles, and heights for each Janus monolayer.

We assessed dynamic stability using Phonopy^28^ with 6x6x1 supercells and a 2x2x1 **k**-points grid based on the finite displacement method, incorporating rotational symmetry of free space using the HiPhive package^29^. We ensured that the out-of-plane acoustic mode (ZA) strictly followed Born–Huang constraints^30^. Mechanical properties were obtained using the Strain-Energy methodology^31^, considering ion relaxation coefficients through the rectangular unit cell, as shown in Fig. 1a. Carrier mobility and relaxation time were derived from the deformation potential theory proposed by Bardeen and Shockley^32^. Ab initio molecular dynamics simulations utilized a 4x4x1 supercell and a $\Gamma$-centered **k**-point mesh sampling, with an energy cutoff of 400 eV within the canonical ensemble NVT, implemented using the VASP computational code^33^.

Electronic transport properties were investigated using the Boltzmann transport equations under the constant relaxation time approximation (CRTA) in BoltzTraP2^34^ with a finer 54x54x1 **k**-point mesh using PBE functional. Lattice thermal conductivity was determined using second-order (harmonic, 6x6x1) and third-order (anharmonic, 3x3x1) interatomic force constants (IFCs) through Phonopy+HiPhive and Phono3py^35^ with 2x2x1 and 4x4x1 **k**-points grids, respectively, under the solution of the linearized Boltzmann transport equation (LBTE)^36^ with a Q–grid of 80x80x1. To account for the influence of lattice conductivity along the *z* axis on 2D materials, a scaling factor was applied by multiplying $L_{z}/h$, where $L_z$ is the length of the unit cell along the *z* axis and *h* is the thickness of the monolayer, considering only the *x* and *y* directions. All thermoelectric properties were evaluated in the temperature range from 300K to 800K.

**Table 1:** The calculated values for various properties of Janus $\gamma$-Pb$_2$XY (X$=$S, Se; Y$=$Se, Te; X$\ne$Y) monolayers: lattice constant *a* (Å), bond lengths *d* (Å), bond angles $\phi$ ($^{\circ }$), thickness *h* (Å), cohesive energy $E_{coh}$ (eV/atom), relaxed ion stiffness constants $C_{ij}$, Young’s modulus $Y_{\text {2D}}$ (Nm$^{-1}$), Poisson’s ratio $\nu$. In the notation, the atoms of the top and bottom layers are denoted by X and Y, respectively.

Monolayer	*a*	$d_{\text {Pb{-}{-}X}}$	$d_{\text {Pb{-}{-}Y}}$	$\phi _{\angle \text {X{-}{-}Pb{-}{-}X}}$	$\phi _{\angle \text {Y{-}{-}Pb{-}{-}Y}}$	$\phi _{\angle \text {Pb{-}{-}Pb{-}{-}Pb}}$	*h*	$E_{coh}$	$C_{11}$	$C_{12}$	$C_{66}$	$Y_{\text {2D}}$	$\nu$
$\gamma$-Pb$_2$SSe	4.10	2.72	2.82	97.95	93.40	65.79	5.80	− 4.10	37.54	9.45	14.05	35.16	0.252
$\gamma$-Pb$_2$STe	4.25	2.75	3.00	101.54	90.45	67.39	5.89	− 3.95	36.61	7.51	14.55	35.07	0.205
$\gamma$-Pb$_2$SeTe	4.31	2.86	3.01	97.92	91.51	68.60	6.00	− 3.84	35.71	12.02	11.85	31.66	0.337

## Results and discussion

### Structural properties

The optimized structures for each Janus $\gamma$-Pb$_2$XY (X$=$S, Se; Y$=$Se, Te; X$\ne$Y) monolayer are depicted in Fig. 1a, conforming to the space group *P*3*m*1 (No. 156). Two unit cells are observed: the unit cell denoted by a solid black line is used for the general calculations of this research, whereas the rectangular dashed unit cell denoted in red is employed for the mechanical properties of the material. Additionally, in Fig. 1b, a vacuum implementation along the *c* direction of 30 Å is shown to eliminate any other periodic interactions and work directly with the *xy* plane.

We also provide the heights, distances, and angles between atoms of each monolayer, as observed in Table 1. Notably, Janus monolayers can be formed by replacing the top part of the $\gamma$-PbX monolayers, substituting the top layer with another chemical element. The obtained lattice parameters fall within the range of 4.10–4.31 Å, as shown in Table 1. These values are consistent with the average values of their base monolayers ($\gamma$-PbX), which are 4.04, 4.18, and 4.43 Å for $\gamma$-PbS, $\gamma$-PbSe, and $\gamma$-PbTe, respectively, as demonstrated in the theoretical work by Jia et al.^10^. This behavior of the lattice parameter for Janus monolayers, being essentially the average of their respective base monolayers, has been observed in other experimental and theoretical results^12,37^. The bond lengths of $d_\text {Pb--X}$ and $d_\text {Pb--Y}$ increase with the atomic radius of the chalcogenides, similarly to the height *h*, as seen in Table 1.

When predicting new materials through theoretical studies, it is crucial to assess their stability, demonstrated here through cohesion energy, phonon dispersion plots, molecular dynamics, and mechanical stability (section of Mechanical properties). First, we evaluate the strength of the chemical bond (cohesive energy) via

2 $$\begin{aligned} E_{coh}=\frac{E_{\text {Total}}-(N_{\text {Pb}}E_{\text {Pb}}+N_\text {X}E_\text {X} +N_\text {Y}E_\text {Y})}{N_{\text {Pb}}+N_\text {X}+N_\text {Y}}~, \end{aligned}$$

where $E_{\text {Total}}$ represents the total energy of the system for each monolayer, $N_{\text {Pb}}$, $N_{\text {X}}$, and $N_{\text {Y}}$ denote the number of atoms in the unit cell for each element, and $E_{\text {Pb}}$, $E_{\text {X}}$, and $E_{\text {Y}}$ represent the individual energy of each atom for Pb, X, and Y. The results are presented in Table 1, showing negative values, indicating greater attraction between the atoms than the energy required to separate them, suggesting energetic stability, similar with other Janus monolayers^38–40^.

**Figure 2:**
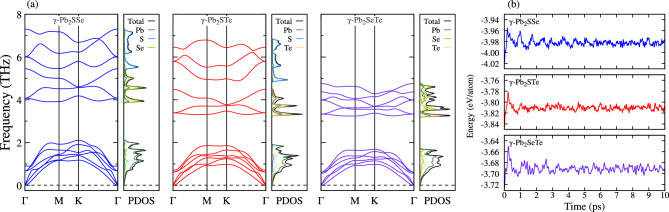
(**a**) The calculated phonon dispersions with their respective atomic projected phonon density of states and (**b**) the results of Ab initio molecular dynamic simulations at 300 K, both for Janus $\gamma$-Pb$_2$XY (X$=$S, Se; Y$=$Se, Te; X$\ne$Y) monolayers.

Additionally, we present the phonon dispersion plot in Fig. 2a, revealing the absence of imaginary frequencies, indicating dynamic stability for each Janus monolayer. Notably, there is no gap between the acoustic and optical phonons, with high-frequency acoustic branches overlapping with low-frequency optical branches. This behavior, observed in other theoretical studies, leads to low thermal conductivity due to strong scattering between acoustic and optical phonons^10,41^. Acoustic phonons are mainly dominated by Pb, while optical phonons are primarily dominated by the top X layer.

We observe that as the atomic mass of the chalcogenides increases, the maximum frequency decreases, with maximum values of 7.31, 6.80, and 4.76 THz for Janus $\gamma$-Pb$_2$SSe, $\gamma$-Pb$_2$STe, and $\gamma$-Pb$_2$SeTe monolayers, respectively. This trend can be attributed to the heavier atoms vibrating at lower frequencies due to their greater inertia, contrasting with lighter atoms that vibrate at higher frequencies. Furthermore, ab–initio molecular dynamics simulations were conducted at room temperature under the canonical NVT ensemble to observe energy fluctuations over a range of 0-10 picoseconds in each Janus monolayer. The energy fluctuation was found to be insignificant, with no observed structural phase changes or chemical bond ruptures during the simulation (Fig. S3 in SM). This result confirms the thermal stability of all Janus monolayers at room temperature, as depicted in Fig. 2b. In addition, according to Bader’s charge analysis (Table S1 in SM), it is evident that Pb$_1$ atoms share 0.712 electrons to X (X=S, Se), being higher compared to the 0.481 electrons shared by Pb$_2$ atoms to Y (Y=Se,Te), so there is a strong bond between Pb$_1$-X and Pb$_2$-Y bonds, gaining more electrons to X and Y atoms in each Janus monolayer due to electronegativity, also forming part of metavalent bonds. This appearance of metavalent bonds in each Janus monolayer corroborates a possible low thermal conductivity at first instance. This same behavior has been seen in the Janus $\gamma$-Ge$_2$SSe^42^ monolayer where its metavalent bond was corroborated by Bader charges.

### Mechanical properties

To confirm the mechanical stability of the Janus monolayers studied here, we assessed the elastic stiffness coefficients $C_{ij}$ (using Voigt notation) utilizing the rectangular unit cell depicted in Fig. 1. We applied uniaxial ($\varepsilon _{x/y}$) and biaxial ($\varepsilon _{xy}$) strains ranging from -1% to 1% with increments of 0.5%, and at each strain level, we performed a relaxation of the atomic positions. The elastic stiffness coefficients were evaluated using the following expressions

3 $$\begin{aligned} \frac{\Delta E(S,\varepsilon _i)}{S_0} = \frac{1}{2}\left( C_{11}\varepsilon _{11}^2 + C_{22}\varepsilon _{22}^2 + 2C_{12}\varepsilon _{11}\varepsilon _{22} + 4C_{66}\varepsilon _{12}^2\right) ~, \end{aligned}$$

4 $$\begin{aligned} C_{11}= \frac{1}{S_0}\frac{\partial U}{\partial \varepsilon _{11}^2},{} & {} C_{22}= \frac{1}{S_0}\frac{\partial U}{\partial \varepsilon _{22}^2},{} & {} C_{12}= \frac{1}{S_0}\frac{\partial U}{\partial \varepsilon _{11}\varepsilon _{22}}~, \end{aligned}$$

where $S_0$ is the area of the rectangular cell, $\Delta E$ is the energy variation with respect to the equilibrium energy, *U* represents the total energy of the system, $\varepsilon _{11}$ is the deformation in the *x* direction, $\varepsilon _{22}$ is the deformation in the *y* direction and $\varepsilon _{12}$ is the simultaneous deformation in the *x* and *y* directions.

To analyze the mechanical properties of the Janus monolayers, we need to obtain the elastic stiffness constants $C_{ij}$ using Voigt notation. Since it is a hexagonal structure, we obtain two elastic stiffness constants, $C_{11}$ and $C_{22}$, along with $C_{12}$, where $C_{66}$ can be derived as $(C_{11} - C_{12})/2$. We organize these constants into a matrix as

5 $$\begin{aligned} C_{ij}= \begin{pmatrix} C_{11} &{} C_{12} &{} 0\\ C_{21} &{} C_{22} &{} 0 \\ 0 &{} 0 &{} (C_{11}-C_{22})/2 \\ \end{pmatrix} ~. \end{aligned}$$

The mechanical stability of these monolayers is determined by Born’s mechanical stability criterion^43^, which requires that $C_{11} > 0$ and $C_{11}^2 > C_{12}^2$. As demonstrated in Table 1, all Janus monolayers in this study meet these criteria, indicating their mechanical stability.

**Figure 3:**
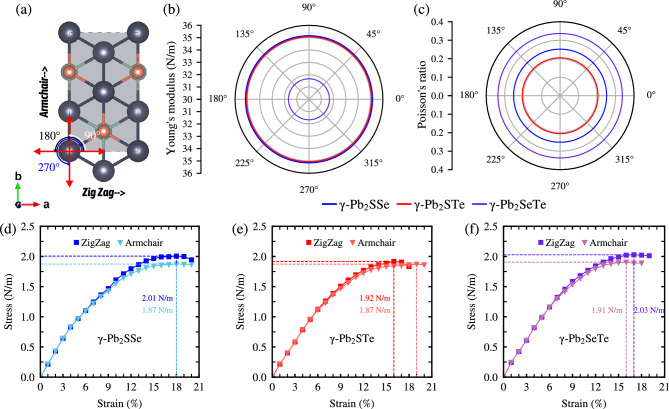
(**a**) Top view of Janus $\gamma$-Pb$_2$XY (X$=$S, Se; Y$=$ Se, Te; X$\ne$Y) monolayers, illustrating the Zigzag and Armchair directions based on the rectangular unit cell. (**b**) Young’s modulus $Y{_{\text {2D}}}(\theta )$, (**c**) Poisson’s ratio $\nu (\theta )$ as functions of the angle $\theta$ and (**d**–**f**) Stress-strain curve along Zig Zag (*x*) and Armchair (*y*) direction.

To examine the angular dependence of Young’s modulus and Poisson’s ratio, we utilize the elastic stiffness coefficients defined in Equation (4). We establish the following relationships

6 $$\begin{aligned} Y_{\text {2D}}(\theta )=\frac{C_{11}C_{22}-C_{12}^2}{C_{11}\text {sin}^4\theta +C_{22}\text {cos}^4\theta -\Pi \text {sin}^2\theta \text {cos}^2\theta }~, \end{aligned}$$

7 $$\begin{aligned} \nu (\theta )= \frac{C_{11}C_{22}- \Pi \text {sin}^2\theta \text {cos}^2\theta - C_{12}(\text {sin}^4\theta +\text {cos}^4\theta )}{C_{11}\text {sin}^4\theta +C_{22}\text {cos}^4\theta +\Pi \text {sin}^2\theta \text {cos}^2\theta }~, \end{aligned}$$

where $\Pi = \left[ (C_{11}C_{22}-C_{12}^2)/C_{66} - 2C_{12} \right]$ and $\theta$ represents the angular dependence for Young’s modulus and Poisson’s coefficient, respectively. As illustrated in Fig. 3 and detailed in Table 1, directional mechanical isotropy is observed for each Janus monolayer, with low Young’s modulus indicating flexibility. Deformation is applied in a given direction to determine the ideal strength and critical strain. This is expressed as $\varepsilon _{x/y,xy} = (a - a_0)/a_0$, where $a_0$ represents the optimized lattice parameter and *a* is the material’s deformed value based on the rectangular unit cell. As seen in Fig. 3d–f, uniaxial deformation is applied in the *x* direction (Zig Zag) and *y* direction (Armchair), meanwhile biaxial deformation along *xy* direction is at Figure S2 in SM. The vacuum through *c* rescaled the stress. It was demonstrated that the stress along the ZigZag direction can handle more stress compared to the Armchair direction, reaching a critical point of 2.01 (1.87) N/m, 1.92 (1.87) N/m, and 2.03 (1.91) N/m at a critical strain of 18 (18) %, 16 (18) %, and 17 (16) % along the ZigZag (Armchair) direction for Janus $\gamma$-Pb$_2$SSe, $\gamma$-Pb$_2$STe, and $\gamma$-Pb$_2$SeTe monolayers, respectively. As observed in Figure S2, the stress resistance is higher compared to the uniaxial ones, reaching a critical point almost twice as high compared to the uniaxial strains, with values of 3.78, 4.87, and 3.70 at a critical strain of 17, 17, and 24 % for Janus $\gamma$-Pb$_2$SSe, $\gamma$-Pb$_2$STe, and $\gamma$-Pb$_2$SeTe monolayers, respectively. Among the studied Janus monolayers, $\gamma$-Pb$_2$STe withstands the highest stress and highest biaxial strain. It is crucial to understand the maximum stress that Janus $\gamma$-Pb$_2$XY (X$=$S, Se; Y$=$ Se, Te; X$\ne$Y) monolayers can withstand, as the material can break once it reaches its maximum strength.

### Electronic properties

**Figure 4:**
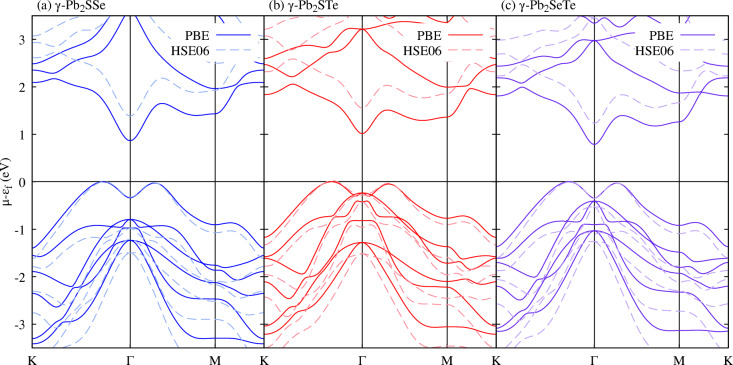
The band structures of Janus $\gamma$-Pb$_2$XY (X$=$S, Se; Y$=$ Se, Te; X$\ne$Y) monolayers, calculated using the PBE and HSE06 functionals. The results are represented by solid and dashed lines, respectively, showcasing the electronic behavior of these materials under different theoretical frameworks, (**a**) Represent the band structure for $\gamma$-Pb$_2$SSe (**b**) Represent the band structure for $\gamma$-Pb$_2$STe (c) Represent the band structure for $\gamma$-Pb$_2$SeTe.

Afterwards, we delved into the electronic characteristics of the Janus $\gamma$-Pb$_2$XY (X$=$S, Se; Y$=$ Se, Te; X$\ne$Y) monolayers, considering both the PBE and HSE06 functionals. The band structure outcomes are illustrated in Fig. 4, revealing that all monolayers possess an indirect bandgap. Specifically, using the PBE functional, the bandgap measures 0.87 eV, 1.01 eV, and 0.78 eV for $\gamma$-Pb$_2$SSe, $\gamma$-Pb$_2$STe, and $\gamma$-Pb$_2$SeTe, respectively. With the HSE06 functional, these values increase to 1.39 eV, 1.53 eV, and 1.23 eV for the same monolayers. These bandgaps exceed those reported for their base monolayers by Nair et al.^41^.

In all Janus monolayers, the conduction band minimum (CBM) is located at the $\Gamma$ point, and the valence band maximum (VBM) is at the K-$\Gamma$ point. Around the VBM, the presence of the Mexican hat is noted, which was also observed in other $\gamma$-monolayers^44–47^. Both peaks are located at K-$\Gamma$ and $\Gamma$-M along with a small energy level difference, which can be influenced by external factors, primarily deformation, resulting in an energy level shift, causing carrier mobility and corresponding bandgaps to be affected^48–51^. In the case of these Janus monolayers in the $\gamma$ phase, due to the new valley at $\Gamma$-M.

**Figure 5:**
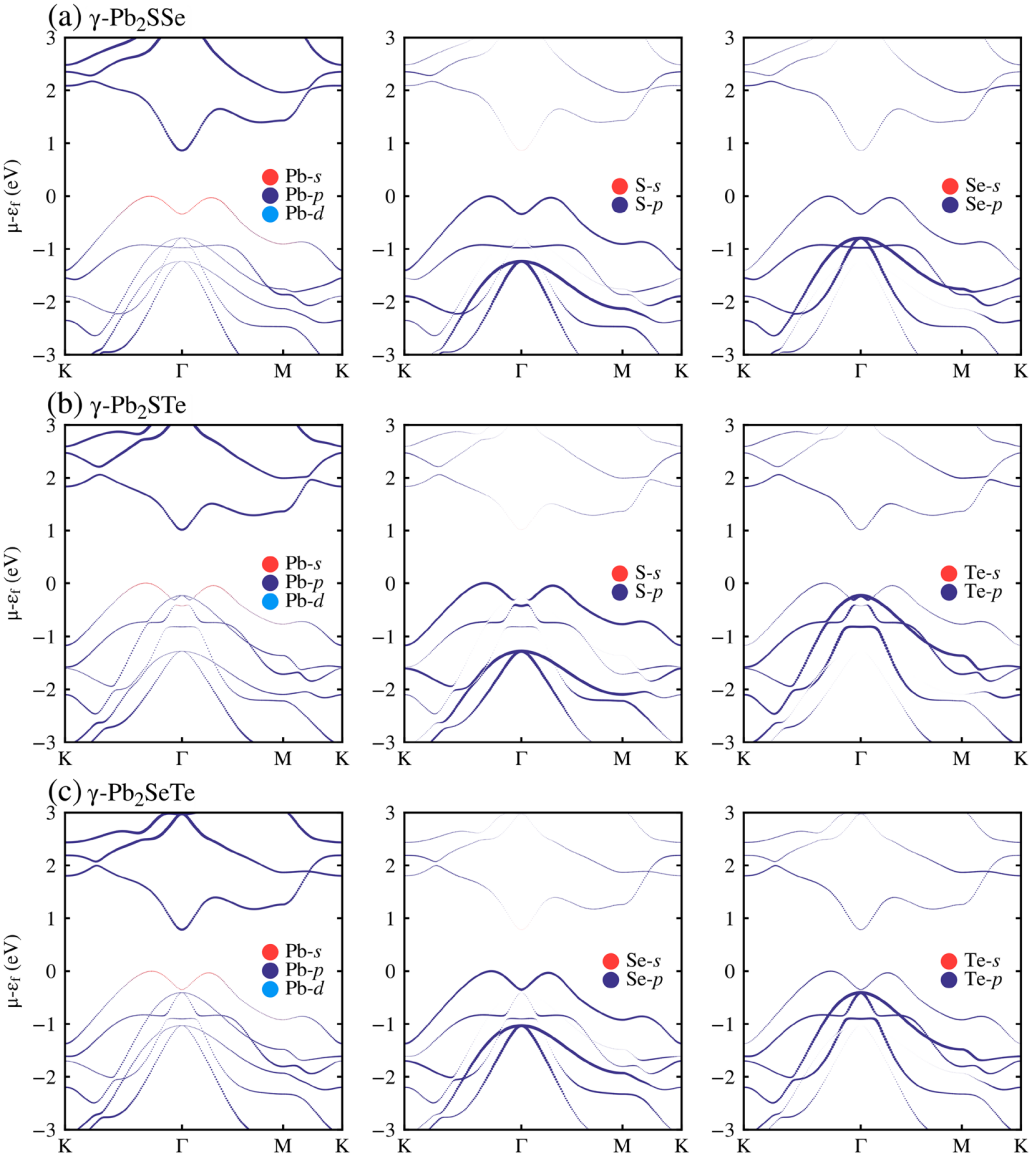
The projected band structure of Janus $\gamma$-Pb$_2$XY (X$=$S, Se; Y$=$ Se, Te; X$\ne$Y) monolayers using the PBE functional. (**a**) Shows the projected band structure for $\gamma$-Pb$_2$SSe. (**b**) Shows the projected band structure for $\gamma$-Pb$_2$STe. (**c**) Shows the projected band structure for $\gamma$-Pb$_2$SeTe.

Next, we evaluated the projected band structure to determine which orbitals of each element play a significant role in CBM and VBM, as shown in Fig. 5. Primarily, it is observed that CBM is mainly dominated by the Pb-*p* orbitals in each Janus monolayer, while VBM is primarily dominated by the X-*p* orbital (the compound of the top layer) with a slight contribution from the Y-*p* orbitals (the compound of the bottom part). This representation provides insights into the electronic properties of the materials, illustrating the contributions of different atomic orbitals to the band structure.

**Figure 6:**
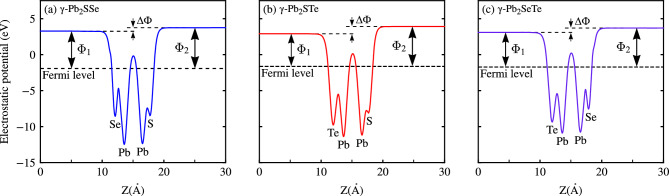
The average plane electrostatic potentials of Janus $\gamma$-Pb$_2$XY (X$=$S, Se; Y$=$ Se, Te; X$\ne$Y) monolayers, along with the work functions on the bottom side Y and top X. This visualization provides insights into the charge distribution and electrostatic properties of the monolayers. Additionally, the work functions $\Phi _1$ and $\Phi _2$ on the bottom and top sides, respectively, are indicated, with $\Delta \Phi$ representing the difference in the vacuum level between the two sides. (**a**) Shows the electrostatic potential and work functions for $\gamma$-Pb$_2$SSe. (**b**) Shows the electrostatic potential and work functions for $\gamma$-Pb$_2$STe. (**c**) Shows the electrostatic potential and work functions for $\gamma$-Pb$_2$SeTe.

Another important parameter to consider in electronic properties is the Work Function, which is basically the energy required for an electron to leave the material surface and can be determined by average planar electrostatic potentials. As can be seen in Fig. 6, where the Work Function is given by $\Phi = E_{\text {vac}} - E_{\text {F}}$, where $E_{\text {vac}}$ is the vacuum energy and $E_{\text {F}}$ is the Fermi level.

Due to the structural asymmetry resulting from the difference in atoms both at the top and bottom of the monolayer, a difference in electronegativity is proposed, necessitating the correction of dipoles on each Janus monolayers. It was confirmed that there is a difference in vacuum energy on both sides of the Janus monolayers (which consequently causes a difference in work functions), with a dependence on atomic radius; the greater the difference in atomic radius between X and Y, the greater their $\Delta \Phi$. The results are observed in Table 2, obtaining $\Delta \Phi$ of 0.47, 1.02, and 0.69 eV for Janus $\gamma$-Pb$_2$SSe, $\gamma$-Pb$_2$STe, and $\gamma$-Pb$_2$SeTe monolayers, respectively. Based on $\Phi _1$ and $\Phi _2$, we can conclude that $\Phi _1$, corresponding to the bottom part of the monolayer, is greater than $\Phi _2$, corresponding to the top part of the monolayer, suggesting that electrons from the top layer are relatively easier to remove compared to $\Phi _1$, consequently requiring less energy to escape from the surface.

**Table 2:** The bandgaps ($E_\text {g}$) calculated using both PBE and HSE06 functionals, along with the vacuum energy (${\bar{E}}_\text {Vac}$), the Fermi level ($E_\text {F}$), the work functions ($\Phi _1$ and $\Phi _2$) with respect to the bottom and top layers, respectively, and their corresponding work function difference ($\Delta \Phi$) are summarized for Janus $\gamma$-Pb$_2$XY (X$=$S, Se; Y$=$ Se, Te; X$\ne$Y) monolayers. All values shown are in eV.

Monolayer	$E_\text {g}^{\text {PBE}}$	$E_\text {g}^{\text {HSE06}}$	${\bar{E}}_\text {Vac}$	$E_\text {F}$	$\Delta \Phi$	$\Phi _1$	$\Phi _2$
$\gamma$-Pb$_2$SSe	0.87	1.39	3.51	− 1.92	0.47	5.19	5.66
$\gamma$-Pb$_2$STe	1.01	1.53	3.41	− 1.64	1.02	4.54	5.56
$\gamma$-Pb$_2$SeTe	0.78	1.23	3.36	− 1.72	0.69	4.73	5.42

### Carrier mobility

Carrier mobility is a crucial parameter in determining the potential applications of materials in electronic devices. In this study, we employed the deformation potential theory proposed by Bardeen and Shockley^32^, which has been widely utilized in theoretical investigations and has been adapted for 2D materials^52^. The carrier mobility ($\mu _{\text {2D}}$) can be expressed as

8 $$\begin{aligned} \mu _{\text {2D}}=\frac{e\hbar ^3C_{\text {2D}}}{k_BTm^*{\overline{m}}E_d^2}~, \end{aligned}$$

where, *e* and $\hbar$ represent the elementary charge and reduced Planck’s constant, respectively. $C_{\text {2D}}$ denotes the 2D elastic stiffness, obtained by fitting the strain-energy curve along the *x*/*y* directions:

9 $$\begin{aligned} C_{\text {2D}}=\frac{1}{A_0} \frac{\partial E_{{\text {s}}}}{\varepsilon _{\text {uni}}^2}~, \end{aligned}$$

where $A_0$ is the equilibrium unit cell area, and $E_s$ is the strain-energy difference between the deformed and undeformed unit cell. Additionally, $k_B$ is Boltzmann constant, *T* is the temperature (room temperature), $m^*$ and ${\overline{m}}$ represent the effective mass and its average, respectively, determined by fitting a parabolic function to the energy dispersion curve for both electrons (CBM) and holes (VBM) along the *x*/*y* transport direction. The effective mass ($m^*$) is given by

10 $$\begin{aligned} m^*=\hbar ^2\left[ \frac{\partial ^2E(k)}{\partial k^2}\right] ^{-1}~, \end{aligned}$$

while the average effective mass (${\overline{m}}$) is calculated as the geometric mean of the masses along the *x* and *y* directions (${\overline{m}}=\sqrt{m_xm_y}$). Furthermore, $E_d$ represents the deformation potential constant for CBM and VBM along the *x*/*y* directions, as defined below

11 $$\begin{aligned} E_d=\frac{\Delta E_{\text {edge}}}{\varepsilon _{\text {uni}}}~, \end{aligned}$$

**Table 3:** The carrier effective mass, $m^*$, 2D elastic modulus, $C_{\text {2D}}$ (Nm$^{-1}$), deformation potential constant, $E_d$ (eV), carrier mobility, $\mu _{\text {2D}}$ (cm$^2$V$^{-1}$s$^{-1}$), and relaxation time, $\tau$ (fs), at room temperature, along the transport directions *x* and *y* for Janus $\gamma$-Pb$_2$XY (X$=$S, Se; Y$=$ Se, Te; X$\ne$Y) monolayers. Here, $m_0$ denotes the mass of a free electron.

	Monolayer	$m_x^*/m_0$	$m_y^*/m_0$	$C^x_{\text {2D}}$	$C^y_{\text {2D}}$	$E^x_d$	$E^y_d$	$\mu ^x_{\text {2D}}$	$\mu ^y_{\text {2D}}$	$\tau _x$	$\tau _y$
Electron	$\gamma$-Pb$_2$SSe	0.17	0.17	24.83	24.81	− 8.486	− 8.486	264.39	264.18	25.05	25.03
	$\gamma$-Pb$_2$STe	0.14	0.14	26.76	26.84	− 8.346	− 8.362	393.56	393.17	32.26	32.23
	$\gamma$-Pb$_2$SeTe	0.13	0.13	29.41	29.41	− 8.962	− 8.962	461.28	461.42	34.10	34.11
Hole	$\gamma$-Pb$_2$SSe	0.55	0.55	24.83	24.81	− 4.174	− 4.170	101.28	101.40	31.52	31.56
	$\gamma$-Pb$_2$STe	0.56	0.56	26.76	26.84	− 4.760	− 4.760	78.85	79.07	25.32	25.39
	$\gamma$-Pb$_2$SeTe	0.47	0.47	29.41	29.41	− 4.906	− 4.906	118.26	119.29	31.54	31.55

where $\Delta E_{\text {edge}}$ is the energy displacement for CBM and VBM concerning the vacuum level when a uniaxial strain $\varepsilon _{x/y}$ is applied.

**Figure 7:**
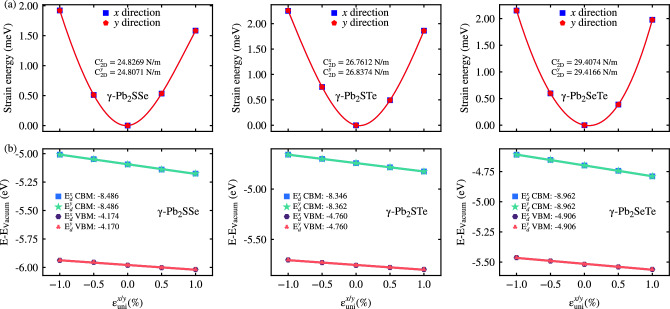
(**a**) Strain-energy and (**b**) CBM/VBM of Janus $\gamma$-Pb$_2$XY (X$=$S, Se; Y$=$ Se, Te; X$\ne$Y) monolayers plotted against the uniaxial strains along the *x*/*y* direction ($\epsilon ^{x/y}_{\text {uni}}$). Data fitting is represented by solid lines in each plot.

The results obtained from applying this theory to determine $C_{\text {2D}}$ and $E_d$ are depicted in Fig. 7, with solid lines representing the fitting performed to obtain these values. Table 3 presents the obtained values, showcasing isotropic behavior for all monolayers in this study. Moreover, it is notable that the carrier mobility for electrons exceeds that of holes, which can be attributed to factors such as effective mass and potential deformation constant. Across all monolayers, the effective mass of electrons is lower than that of holes, a significant factor in electronic devices as it implies greater curvature in CBM/VBM, facilitating a better response to charge carriers. Additionally, the potential deformation constant is relatively lower in VBM compared to CBM due to a low slope, contributing to a carrier mobility that is not excessively low. For instance, the carrier mobility for electrons (holes) was observed to be 264.39 (101.28), 393.56 (78.85), and 461.42 (118.26) cm$^2$V$^{-1}$s$^{-1}$ along the X-direction, with a similar trend observed along the Y-direction, for Janus $\gamma$-Pb$_2$SSe, $\gamma$-Pb$_2$STe, and $\gamma$-Pb$_2$SeTe monolayers, respectively (as shown in Table 3).

It is worth noting that all Janus monolayers in this study exhibit high carrier mobility, surpassing that of single-layer MoS$_2$ with a value of 200 cm$^2$V$^{-1}$s$^{-1}$ reported by Radisavljevic et al.^53^. However, with increasing temperature, the carrier mobility performance tends to decrease (see Fig. S4), attributed to the rise in phonon contributions. Nevertheless, it is important to emphasize that using the DP theory overestimates the carrier mobility because it does not include different mechanisms of carrier scattering given mainly by electron-phonon couplings.

### Phonon transport properties and lattice thermal conductivity

The lattice thermal conductivity, determined via the LBTE, is expressed using Cartesian coordinates $\alpha \beta$ as follows

$$\begin{aligned} \kappa ^{\alpha \beta }={} & {} \sum _b\frac{1}{k_{\text {B}}T^2V} \times \langle f{_0}(\omega _{{{\textbf {q}}},b};T)[1+f_{0}(\omega _{{{\textbf {q}}},b};T)](\hbar \omega _{{{\textbf {q}}},b})^2 \upsilon _{{{\textbf {q}}},b}^{\alpha }F_{{{\textbf {q}}},b}^{\beta }\rangle _{{{\textbf {q}}}}~. \end{aligned}$$

Here, *b* represents a phonon mode, $\omega _{{{\textbf {q}}},b}$ is the angular frequency, $\upsilon _{{{\textbf {q}}},b}$ is the group velocity for each phonon mode at the ${{\textbf {q}}}$-point in reciprocal space, $f_{0}(\omega _{{{\textbf {q}}},b};T)$ is the equilibrium occupation for a respective phonon mode at temperature *T*, $F_{{{\textbf {q}}},b}$ is the Cartesian vector of coefficients, $\langle \cdots \rangle _{{{\textbf {q}}}}$ denotes averaging over the first Brillouin zone. $F_{{{\textbf {q}}},b}$ is obtained from the solution of the LBTE and is directly related to the phonon lifetime^54^. The group velocity $\upsilon _{{{\textbf {q}}},b}$ and phonon lifetime $\tau _b$ are given by the following relations

12 $$\begin{aligned} \upsilon _{{{\textbf {q}}},b}=\frac{\partial \omega _{b}}{\partial {{\textbf {q}}}} ,{} & {} \tau _b=\frac{1}{2\Gamma _b(\omega _b)}~, \end{aligned}$$

respectively, where the properties of harmonic phonons for each phonon mode *b* are distributed, and $\Gamma _b(\omega _b)$ takes the form analogous to the Fermi golden rule.

Another parameter indicating strong anharmonicity is the Grüneisen parameter $\gamma$, expressed in terms of the reciprocal wave vector ${{\textbf {q}}}$ and the phonon mode *b* through

13 $$\begin{aligned} \gamma _{b}({{\textbf {q}}})=-\frac{a}{\omega _b}\frac{\partial b}{\partial a}~, \end{aligned}$$

Here, *a* is the lattice parameter. The Grüneisen parameter $\gamma$ describes the anharmonicity of a system; the larger $\overline{|\gamma |}$, the stronger the phonon-phonon anharmonic dispersion, leading to low lattice thermal conductivity due to its inverse relationship with the lattice conductivity^55^.

**Figure 8:**
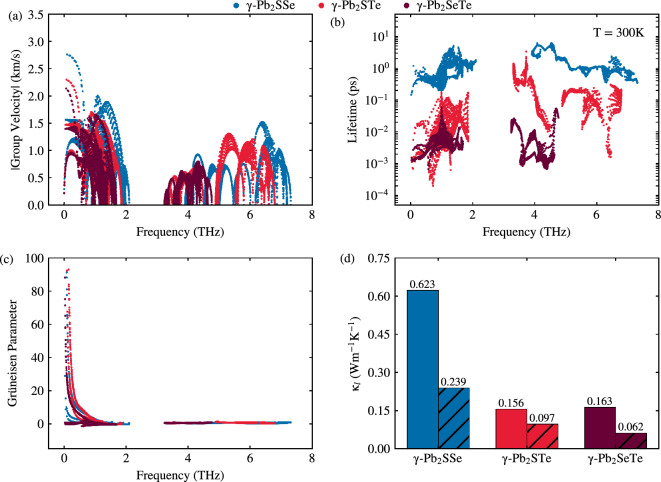
(**a**) Group velocity, (**b**) phonon lifetime, (**c**) Grüneisen parameter, and (**d**) lattice thermal conductivity at 300 K (800 K) with solid filled (dashed) lines for Janus $\gamma$-Pb$_2$XY (X$=$S, Se; Y$=$ Se, Te; X$\ne$Y) monolayers.

To gain deeper insights into the phonon thermal transport mechanisms within each Janus monolayer, we conducted calculations for the group velocity, phonon lifetime, and Grüneisen parameter. Analyzing the group velocity depicted in Fig. 8a, we observe consistently low average values across all Janus monolayers: 0.59, 0.53, and 0.44 km/s for Janus $\gamma$-Pb$_2$SSe, $\gamma$-Pb$_2$STe, and $\gamma$-Pb$_2$SeTe monolayers, respectively. These values are notably lower compared to their primary phases, such as $\gamma$-PbSe^56^, which boasted an average of 1.35 km/s.

The Janus $\gamma$-Pb$_2$SSe monolayer exhibits a distinctive trend, showing higher group velocities in acoustic modes at low frequencies and optical modes at higher frequencies, consistent with the phonon dispersion plot depicted in Fig. 2a. Additionally, we note that the group velocity of acoustic modes surpasses that of optical modes (refer to Fig. S5 in the SM), suggesting a slightly greater contribution from acoustic modes to the overall group velocity.

Understanding the role of group velocity in lattice thermal conductivity ($\kappa _l$) is crucial, given its direct correlation. Lower group velocities are imperative for achieving relatively low $\kappa _l$. However, relying solely on group velocity to understand the behavior of $\kappa _l$ across each Janus monolayer is insufficient. Hence, we further explore other parameters to gain a comprehensive understanding of the observed strong anharmonicity evident in the phonon dispersion plot shown in Fig. 2a.

In Fig. 8b, we visualize the phonon lifetime for each Janus monolayer, where optical modes at higher frequencies demonstrate longer lifetimes, contributing significantly to lattice conductivity. Generally, a low phonon lifetime indicates lower lattice thermal conductivity ($\kappa _l$), as it is inversely related to the anharmonic scattering rate ($\tau _{anh}^{-1}$). Therefore, observing an overall low phonon lifetime across all modes suggests relatively low $\kappa _l$.

Moving on to the Grüneisen parameter depicted in Fig. 8c, we note that ZA acoustic modes exhibit higher anharmonicity at lower frequencies compared to higher-frequency optical modes. This anharmonicity restricts the passage of phonons, resulting in reduced lattice thermal conductivity. Specifically, we obtained Grüneisen parameter values of 0.97, 1.40, and 1.09 for Janus $\gamma$-Pb$_2$SSe, $\gamma$-Pb$_2$STe, and $\gamma$-Pb$_2$SeTe monolayers, respectively. Notably, $\gamma$-Pb$_2$STe displays strong anharmonicity, comparable to well-known thermoelectric materials like PbTe^57^ and CoSb$_3$^58^, which possess Grüneisen parameters around $\sim 1.4$ and $\sim 0.95$, respectively.

All the prerequisites for achieving low lattice conductivity are evident across these factors. However, before calculating lattice thermal conductivity, we must ensure the implementation of rotational invariance conditions to obtain the out-of-plane mode (ZA) strictly quadratic near the gamma point. This requirement is addressed by applying the rotational sum methodology proposed by Born–Huang^30^. Failure to implement this methodology could lead to significant alterations in the results of lattice thermal conductivity, given the sensitivity of phonon modes to even minor changes^59,60^.

The computed values for $\kappa _{l}$ at room temperature are 0.623, 0.156, and 0.163 Wm$^{-1}$K$^{-1}$ for Janus $\gamma$-Pb$_2$SSe, $\gamma$-Pb$_2$STe, and $\gamma$-Pb$_2$SeTe monolayers, respectively (Fig. 8d with solid fill). These values are notably low compared to other gamma phase monolayers such as $\gamma$-GeSe^41,61^ or $\gamma$-PbSe^10,56^ from previous theoretical studies. The achievement of such low $\kappa {_l}$ values is a significant milestone in realizing high thermoelectric performance. Although we consider the quadratic ZA mode for the monolayers in this study, it is also feasible to mention that we have only considered the 3 phonon process involved because we work with the third order anharmonic interatomic forces, being influential also the fourth order anharmonic process when there is a gap between the modes of the phonons and in consequence, a more exact knowdledge of phonon transports compared to three-phonon process.

### Thermoelectric properties

**Figure 9:**
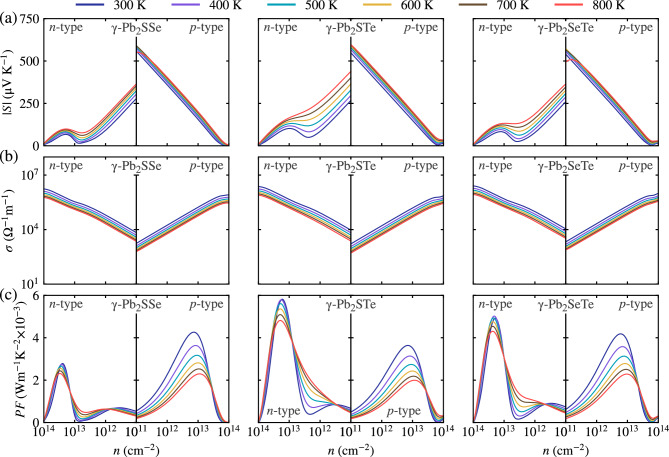
The thermoelectric properties of Janus $\gamma$-Pb$_2$XY (X$=$S, Se; Y$=$ Se, Te; X$\ne$Y) monolayers, focusing on three key parameters: (**a**) Seebeck coefficients, (**b**) electronic conductivity, and (**c**) power factor. These properties are analyzed across different temperatures and carrier concentrations (*n*) to understand the thermoelectric performance of the material comprehensively.

To delve into the thermoelectric properties of Janus $\gamma$-Pb$_2$XY (X$=$S, Se; Y$=$ Se, Te; X$\ne$Y) monolayers, we need to consider crucial parameters that indicate a material’s suitability for thermoelectric applications. The figure of merit ZT, often used for this purpose, is decomposed into the power factor and the total lattice contribution (comprising electronic $\kappa _e$ and lattice $\kappa _l$ contributions), as shown in Eq. (1). In the results discussed below, we consider both *n*-type ($n < 0$) and *p*-type ($n > 0$) behaviors, corresponding to electrons and holes, respectively, across temperatures ranging from 300 K to 800 K, with increments of 100 K. Since BoltzTraP2 determines the electrical conductivity and electronic contribution assuming a constant relaxation time, it is essential to obtain $\tau$ as it varies for each material. In this study, we utilized the deformation potential theory to determine $\tau$, as shown in Equation 14,

14 $$\begin{aligned} \tau =\frac{\mu _{\text {2D}}m^*}{e}~, \end{aligned}$$

where the values of $\tau$ at room temperature are listed in Table 3. Moreover, the absolute values of the Seebeck coefficient for *n*-type and *p*-type carrier concentrations are shown in Fig. 9a. Notably, the Seebeck coefficient for *p*-type carriers is higher compared to *n*-type, accompanied by a reduction in the Seebeck coefficient with increasing carrier concentration. This behavior can be described by considering Mott’s relationship^62^,

15 $$\begin{aligned} S=\frac{8\pi ^2 k_B^2T}{3eh^2}m^*\left( \frac{\pi }{3n}\right) ^{\frac{2}{3}}~, \end{aligned}$$

the $m^*$ and *n* values significantly influence the Seebeck coefficient, as illustrated in Table 3. For holes, the effective mass is higher compared to electrons, resulting in a higher Seebeck coefficient that decreases with increasing carrier concentrations. However, for *n*-type carriers, a slight increase in the Seebeck coefficient is observed at higher carrier concentration levels. This behavior arises from the BoltzTraP2 methodology, which operates on the rigid band approximation (RBA) and derives transport coefficients based on the band structure, including contributions from bands away from the Fermi level.

The corresponding values of |*S*| concerning the optimal *ZT* value at room temperature are presented in Table 4, with 247.1 (196.9), 289.9 (240.1), and 301.6 (242.1) $\upmu$VK$^{-1}$ for Janus $\gamma$-Pb$_2$SSe, $\gamma$-Pb$_2$STe, and $\gamma$-Pb$_2$SeTe monolayers of *p* (*n*)-type, respectively. Particularly, the *p*-type Seebeck coefficients stand out as indicators of good thermoelectric materials, consistent with other excellent thermoelectric materials^58,63^.

On the other hand, the electrical conductivity exhibits a linear behavior with increasing carrier concentration, as depicted in Fig. 9b. The variation between charge carriers can be attributed to the following relationship

16 $$\begin{aligned} \sigma =\frac{ne^2\tau }{m^*}~, \end{aligned}$$

where a high charge carrier mobility depends on a low effective mass and higher carrier concentration. This dependency benefits *n*-type electron carriers more compared to *p*-type carriers.

Concerning the optimal *ZT* value, the electrical conductivity at room temperature was determined for *p* (*n*)-type, resulting in values of 63276 (17612), 31618 (13015), and 32865 (13351) $\Omega ^{-1}$m$^{-1}$ for Janus $\gamma$-Pb$_2$SSe, $\gamma$-Pb$_2$STe, and $\gamma$-Pb$_2$SeTe monolayers, respectively (see Table 4). These values indicate good electrical conductivities for both charge carriers, further supporting the thermoelectric potential of these Janus monolayers.

Furthermore, Fig. 9c depicts the power factor as a function of carrier concentration at different temperatures, providing insight into the thermoelectric performance. The power factor balances the opposing trends of the Seebeck coefficient and electrical conductivity, offering an indication of thermoelectric efficiency. It is notable that the power factor initially increases with rising carrier concentration and then decreases, a trend observed across all Janus monolayers for *p*-type carrier concentrations. However, for *n*-type carriers, two peaks are observed, corresponding to the slight increase in the Seebeck coefficient at high charge carrier concentrations. This behavior is reflected in the *n*-type power factor. Remarkably, the power factor values concerning the optimum *ZT* value for *p*(*n*) type are 3.863 (0.683), 2.657 (0.750), and 2.989 (0.782) Wm$^{-1}$K$^{-2}\times 10^{-3}$ for Janus $\gamma$-Pb$_2$SSe, $\gamma$-Pb$_2$STe, and $\gamma$-Pb$_2$SeTe monolayers, respectively (see Table 4). These values exceed those of PbTe^64^, a material recognized for its excellent thermoelectric properties.

**Table 4:** The calculated optimal dimensionless figure-of-merit (*ZT*) and the corresponding Seebeck coefficient |*S*| ($\upmu$VK$^{-1}$), electronic conductivity $\sigma$ ($\Omega ^{-1}$m$^{-1}$), power factor *PF* (Wm$^{-1}$K$^{-2}\times 10^{-3}$), electronic thermal conductivity $\kappa _e$ (Wm$^{-1}$K$^{-1}$), and carrier concentration *n* (cm$^{-2}$) of *n*-type and *p*-type for Janus $\gamma$-Pb$_2$XY (X$=$S, Se; Y$=$ Se, Te; X$\ne$Y) monolayers at 300 and 800 K.

*T*	Type	Monolayer	|*S*|	$\sigma$	*PF*	$\kappa _e$	*ZT*	*n*
300	*n*-type	$\gamma$-Pb$_2$SSe	196.9	17612	0.683	0.083	0.29	$2.6 \times 10^{11}$
		$\gamma$-Pb$_2$STe	240.1	13015	0.750	0.060	1.04	$1.4 \times 10^{11}$
		$\gamma$-Pb$_2$SeTe	242.1	13351	0.782	0.061	1.05	$1.2 \times 10^{11}$
	*p*-type	$\gamma$-Pb$_2$SSe	247.1	63276	3.863	0.232	1.36	$3.6 \times 10^{12}$
		$\gamma$-Pb$_2$STe	289.9	31618	2.657	0.114	2.96	$1.8 \times 10^{12}$
		$\gamma$-Pb$_2$SeTe	301.6	32865	2.989	0.116	3.22	$1.5 \times 10^{12}$
800	*n*-type	$\gamma$-Pb$_2$SSe	253.8	8322	0.536	0.107	1.24	$3.8 \times 10^{11}$
		$\gamma$-Pb$_2$STe	369.6	5446	0.744	0.119	2.76	$2.3 \times 10^{11}$
		$\gamma$-Pb$_2$SeTe	325.8	5399	0.573	0.099	2.85	$1.7 \times 10^{11}$
	*p*-type	$\gamma$-Pb$_2$SSe	297.2	19693	1.739	0.157	3.52	$3.4 \times 10^{12}$
		$\gamma$-Pb$_2$STe	339.9	10543	1.218	0.086	5.33	$2.7 \times 10^{12}$
		$\gamma$-Pb$_2$SeTe	357.3	9828	1.255	0.084	6.88	$1.4 \times 10^{12}$

The electronic thermal contribution ($\kappa _e$), as shown in Fig. S6, exhibits a linear increase with increasing carrier concentration, similar to the behavior observed for electrical conductivity. This correlation can be attributed to the Wiedemann-Franz law^65^, indicating that *p*-type carriers are favored due to their lower electronic contribution compared to *n*-type carriers.

The optimal values of the figure of merit *ZT* were obtained at room temperature, with electronic contributions for *p* (*n*)-type of 0.232 (0.083), 0.114 (0.060), and 0.116 (0.061) Wm$^{-1}$K$^{-1}$ for Janus $\gamma$-Pb$_2$SSe, $\gamma$-Pb$_2$STe, and $\gamma$-Pb$_2$SeTe monolayers, respectively. This relatively low electronic contribution is crucial for thermoelectric applications as it influences $\kappa _{\text {Tot}}$, where $\kappa _{\text {Tot}} = \kappa _e + \kappa _{l}$.

**Figure 10:**
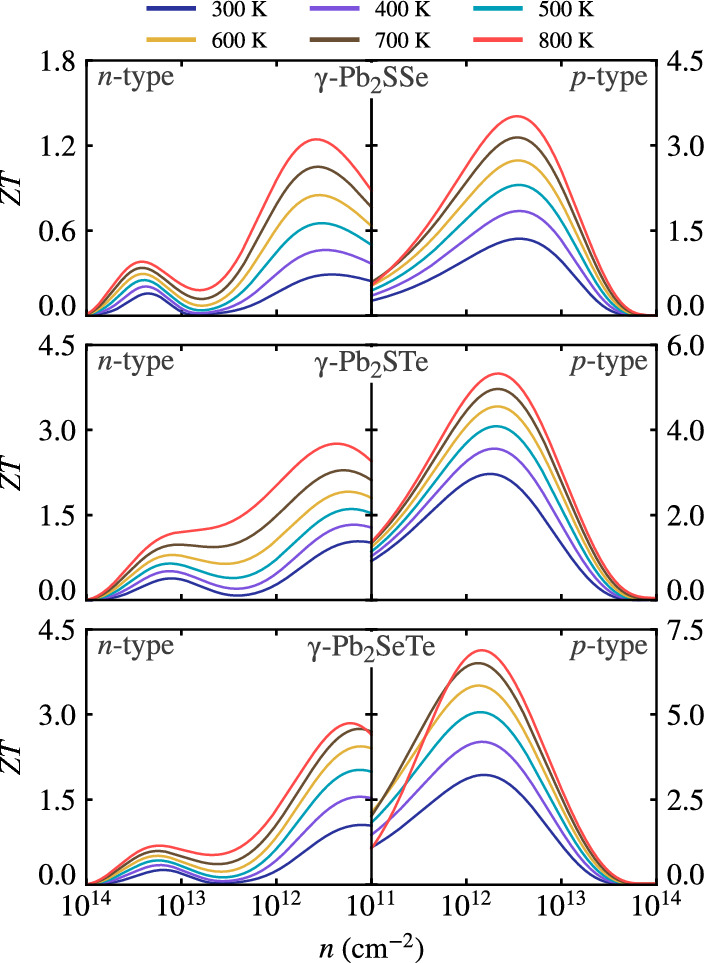
The figure of merit (ZT) as a function of carrier concentration for Janus $\gamma$-Pb$_2$XY (X$=$S, Se; Y$=$ Se, Te; X$\ne$Y) monolayers.

Based on the high power factor and low lattice conductivity, superior thermoelectric performance is expected, which can be quantified by the figure of merit *ZT*. Figure 10 illustrates the *ZT* values as a function of carrier concentration for *n*-type and *p*-type Janus monolayers at different temperatures. Additionally, Table 4 lists the calculated optimal *ZT* values and their corresponding transport properties at room temperature. Clearly, the *ZT* values for each Janus monolayer first increase and then decrease with increasing carrier concentration. For *n*-type Janus monolayers, the optimal carrier concentration ranges between 10$^{10}$–10$^{12}$ cm$^{-2}$, while for *p*-type, it ranges between 10$^{11}$–10$^{13}$ cm$^{-2}$, as shown in Fig. 10. At room temperature, the optimum *ZT* values of *n*-type Janus $\gamma$-Pb$_2$SSe, $\gamma$-Pb$_2$STe, and $\gamma$-Pb$_2$SeTe monolayers reach maximum values of 0.29, 1.04, and 1.05, respectively, with corresponding carrier concentrations of $\sim$ 2.6 $\times$ 10$^{11}$, $\sim$ 1.4 $\times$ 10$^{11}$, and $\sim$ 1.2 $\times$ 10$^{11}$ cm$^{-2}$. Similarly, the optimal *ZT* values of *p*-type Janus monolayers are 1.36, 2.96, and 3.22, with carrier concentrations of $\sim$ 3.6 $\times$ 10$^{12}$, $\sim$ 1.8 $\times$ 10$^{12}$, and $\sim$ 1.5 $\times$ 10$^{12}$ cm$^{-2}$, respectively. Upon reaching 800 K, the optimum *ZT* values for Janus $\gamma$-Pb$_2$SSe, $\gamma$-Pb$_2$STe, and $\gamma$-Pb$_2$SeTe monolayers for *p* (*n*)-type are 3.52 (1.24), 5.33 (2.76), and 6.88 (2.85) at carrier concentrations of 3.4 $\times$ 10$^{12}$ (3.8 $\times$ 10$^{11}$), 2.7 $\times$ 10$^{12}$ (2.3 $\times$ 10$^{11}$), and 1.4 $\times$ 10$^{12}$ (1.7 $\times$ 10$^{11}$) cm$^{-2}$, respectively, showcasing excellent *p*-type thermoelectric performance across all Janus monolayers. These results are higher than those obtained by Ji et al.^10^ without Janus, where they obtained a maximum ZT of 2.45 and higher than other gamma-phase monolayers^9,42,56,66,67^. Nevertheless, it is necessary to note that the thermoelectric properties have been obtained using the PBE functionals without consideration of SOC (BoltzTraP2 does not support SOC in QE), which may alter the thermoelectric properties of some monolayers^68–70^.

## Conclusions

Based on the density functional and deformation potential theories, as well as the Boltzmann transport equation, we have theoretically studied the structural, electronic, transport, phonon, and thermoelectric properties of Janus $\gamma$-Pb$_2$XY (X$=$S, Se; Y$=$ Se, Te; X$\ne$Y) monolayers. We have confirmed the energetic, dynamic, thermal, and mechanical stability of Janus monolayer by applying the Born stability criterion to analyze the cohesion energy, phonon dispersion, and molecular dynamics. We have found that: (i) The electron mobility of these Janus monolayers is isotropic and ranges from 264.18 to 461.42 cm$^2$V$^{-1}$s$^{-1}$, which makes them suitable for the development of nanoelectronic devices. (ii) The high phonon dispersion rates of Janus $\gamma$-Pb$_2$SSe, $\gamma$-Pb$_2$STe, and $\gamma$-Pb$_2$SeTe monolayers at room temperature lead to low lattice thermal conductivities of 0.623, 0.156, and 0.163 Wm$^{-1}$K$^{-1}$, respectively. (iii) A figure of merit *ZT* as high as 3.52, 5.33, and 6.88 were determined for *p*-type carriers. The obtained results thus show the great potential of Janus $\gamma$-Pb$_2$XY (X$=$S, Se; Y$=$ Se, Te; X$\ne$Y) monolayers for thermoelectric applications.

### Supplementary Information

Supplementary Information.

## Data Availability

All data relevant to this study are presented in the manuscript, and its supplementary information files. Any remaining questions or requests for additional details regarding the datasets should be directed to the corresponding author.
