# Supplementary material for: Novel Janus gamma-Pb2XY monolayers with high thermoelectric performance X=S, Se and Y=Se, Te X ≠ Y
Source: Sci Rep. 2024 Jul 19;14:16648. doi: 10.1038/s41598-024-67039-0 (PMC11258366; doi:10.1038/s41598-024-67039-0)
Supplement: Supplementary file 1 — Supplementary Information. [file 41598_2024_67039_MOESM1_ESM.pdf]

Supplementary Material: High Thermoelectric Performance of Novel Janus γ-Pb_2XY (X=S, Se; Y= Se, Te; X≠Y) Monolayers

Efracio Mamani Flores[a,1,**], Victor José Ramirez Rivera[a,1,*], Fredy Mamani Gonzalo[a], Jose Ordonez-Miranda[c], Julio R. Sambrano[d], Mario Lucio Moreira[b], Maurício Jeomar Piotrowski[b]

[a]*Department of Physics, Jorge Basadre Grohmann National University, Tacna, Perú*
[b]*Department of Physics, Federal University of Pelotas, Pelotas, Rio Grande do Sul, Brazil*
[c]*LIMMS, CNRS-IIS IRL 2820, The University of Tokyo, Tokyo 153-8505, Japan*
[d]*Modeling and Molecular Simulation Group, São Paulo State University, Bauru, São Paulo 17033-360, Brazil*

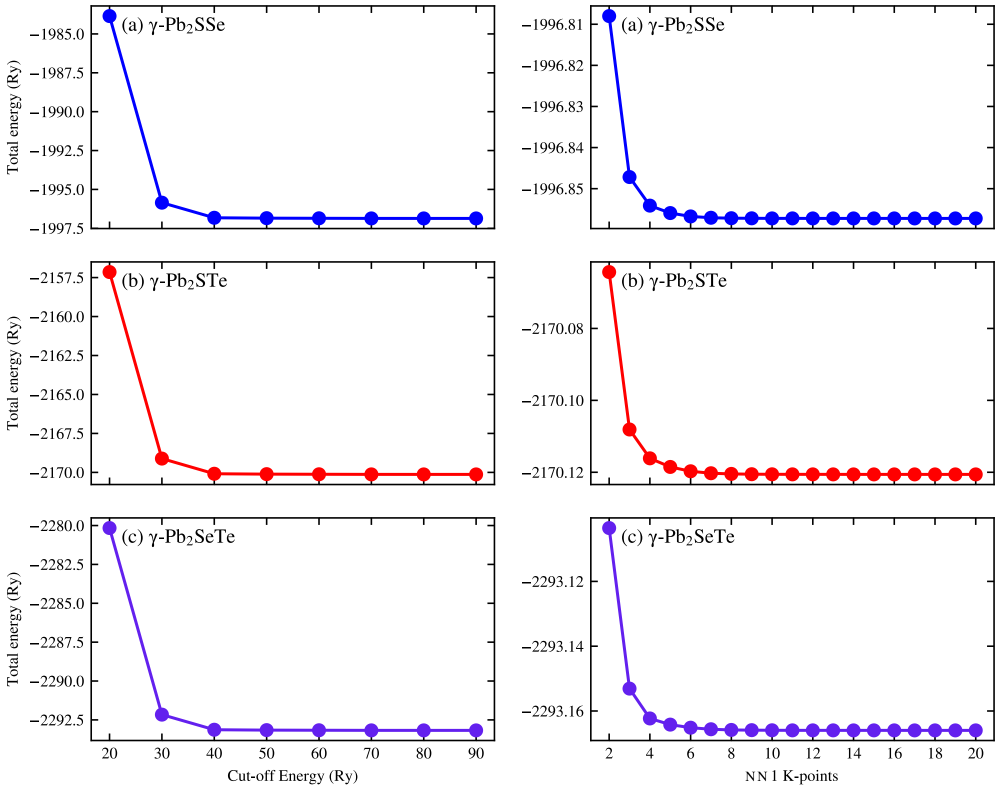

Fig. S1: Convergence tests: Cut-off energy (E_{cut}) and **k**-points (**k**-points N×N×1) for Janus γ-Pb_2XY (X=S, Se; Y= Se, Te; X≠Y) monolayers.

*Corresponding author
**Principal corresponding author
Email addresses: emamanif@unjbg.edu.pe (Efracio Mamani Flores), vramirezr@unjbg.edu.pe (Victor José Ramirez Rivera)

Table S1: Bader charge for Janus γ-Pb_2XY (X=S, Se; Y= Se, Te; X≠Y) monolayers. Pb1 and Pb2 represents the atom of Pb at the top and bottom, respectively, for each Janus monolayer.

Monolayer	Atom	Bader charge (e)
γ-Pb_2SSe	Pb1	0.7390
	Pb2	0.5982
	S	-0.7421
	Se	-0.5951
γ-Pb_2STe	Pb1	0.7637
	Pb2	0.4197
	S	-0.7752
	Te	-0.4081
γ-Pb_2SeTe	Pb1	0.6291
	Pb2	0.4250
	Se	-0.6345
	Te	-0.4193

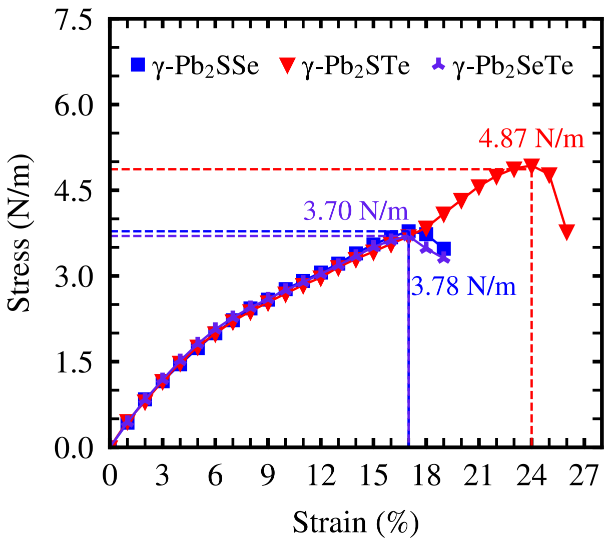

Fig. S2: Stress-Strain curve for Janus γ-Pb_2XY (X=S, Se; Y= Se, Te; X≠Y) monolayers under biaxial strain.

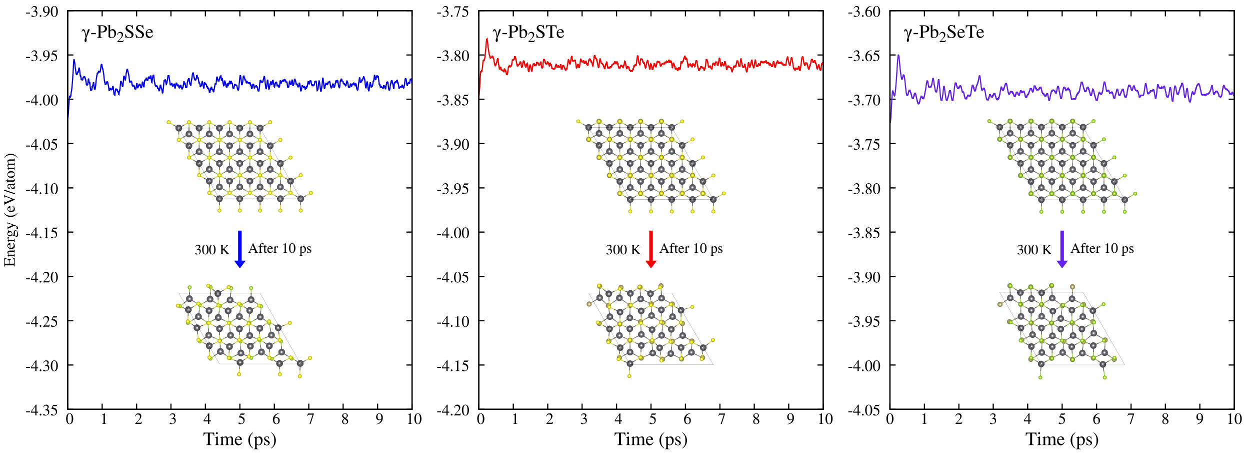

Fig. S3: Ab-initio molecular dynamics (AIMD) for Janus γ-Pb_2XY (X=S, Se; Y= Se, Te; X≠Y) monolayers at 300 K.

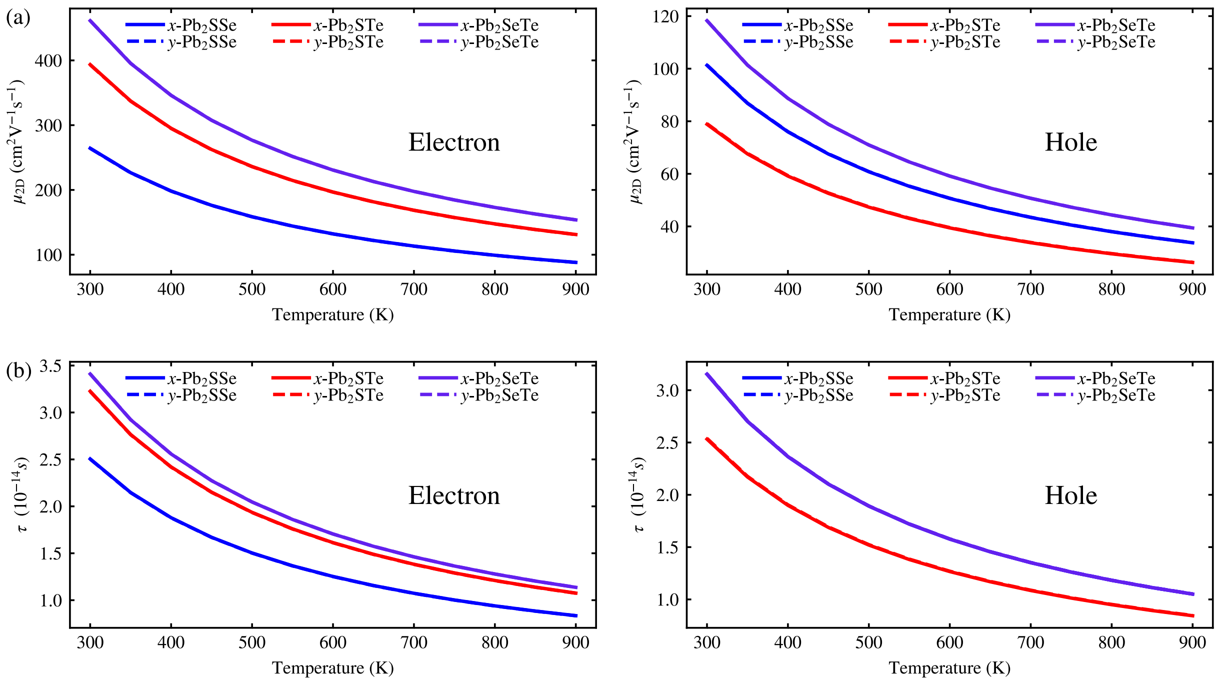

Fig. S4: (a) Carrier mobility and (b) relaxation time as a function of temperature for electrons and holes along x and y direction for Janus γ-Pb_2XY (X=S, Se; Y= Se, Te; X≠Y) monolayers.

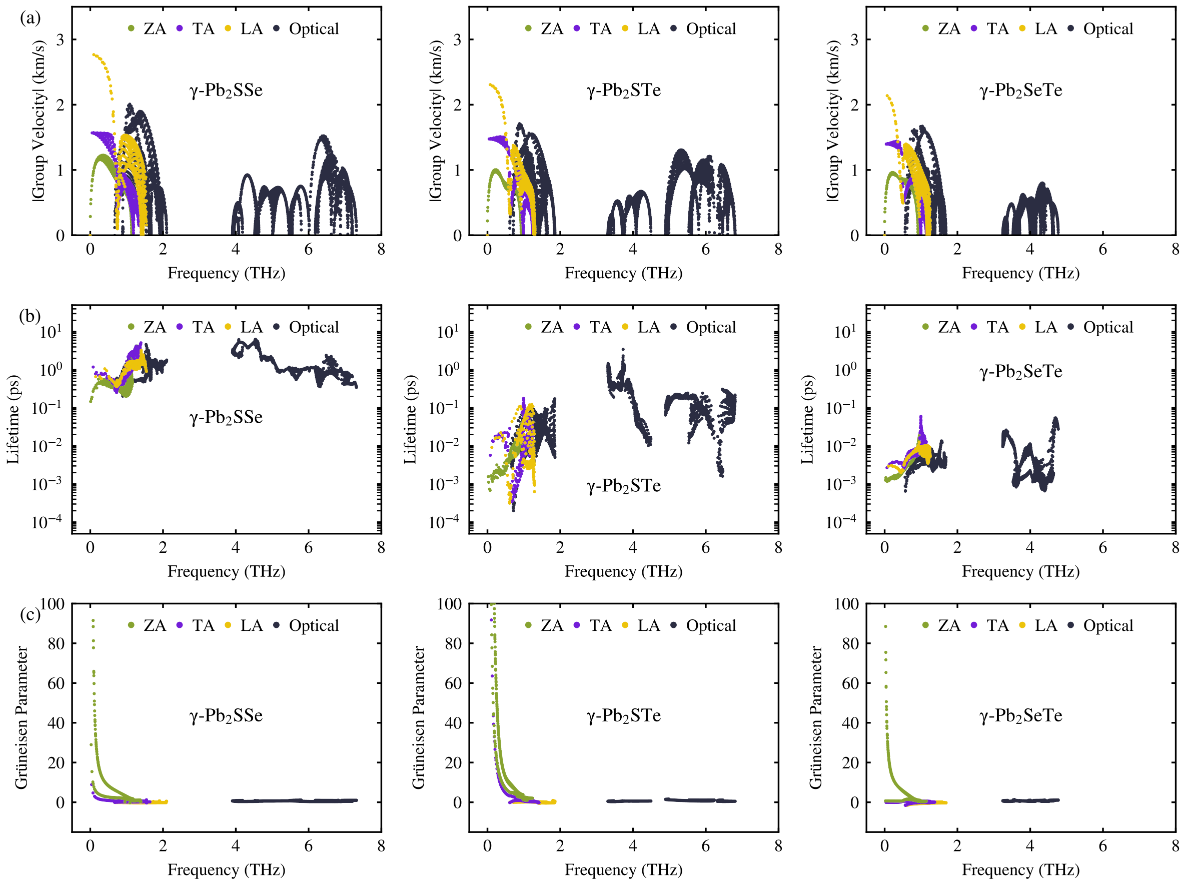

Fig. S5: (a) Group velocity, (b) phonon lifetime and (c) Grüneisen parameter for acustic (ZA, TA and LA) and optical phonon modes b for Janus γ-Pb_2XY (X=S, Se; Y= Se, Te; X≠Y) monolayers.

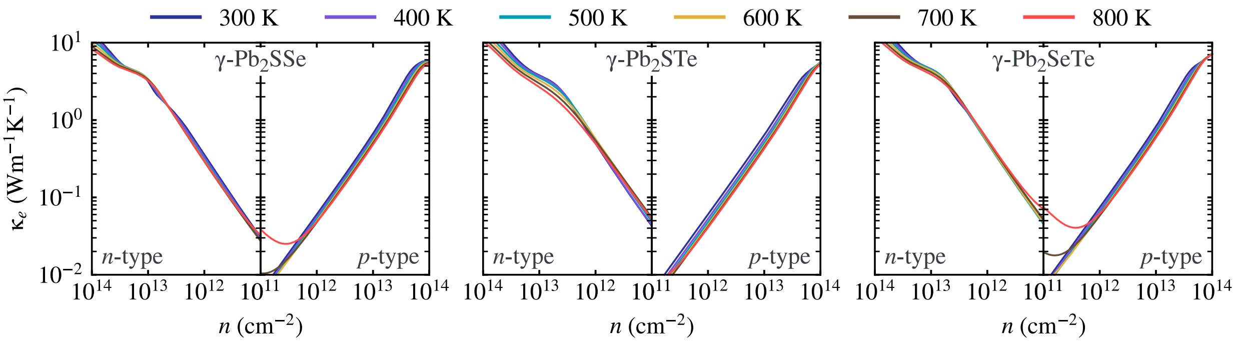

Fig. S6: Electronic thermal contribution κ_e as a function of carrier concentration for Janus γ-Pb_2XY (X=S, Se; Y= Se, Te; X≠Y) monolayers.
